# Deep Learning in Large and Multi-Site Structural Brain MR Imaging Datasets

**DOI:** 10.3389/fninf.2021.805669

**Published:** 2022-01-20

**Authors:** Mariana Bento, Irene Fantini, Justin Park, Leticia Rittner, Richard Frayne

**Affiliations:** ^1^Electrical and Software Engineering, Schulich School of Engineering, University of Calgary, Calgary, AB, Canada; ^2^Hotchkiss Brain Institute, University of Calgary, Calgary, AB, Canada; ^3^Calgary Image Processing and Analysis Centre, Foothills Medical Centre, Calgary, AB, Canada; ^4^School of Electrical and Computer Engineering, University of Campinas, Campinas, Brazil; ^5^Radiology and Clinical Neurosciences, Cumming School of Medicine, University of Calgary, Calgary, AB, Canada; ^6^Seaman Family MR Research Centre, Foothills Medical Centre, Calgary, AB, Canada

**Keywords:** multi-site datasets, deep learning, domain adaptation, data aggregation, batch effects, machine learning, MR brain imaging

## Abstract

Large, multi-site, heterogeneous brain imaging datasets are increasingly required for the training, validation, and testing of advanced deep learning (DL)-based automated tools, including structural magnetic resonance (MR) image-based diagnostic and treatment monitoring approaches. When assembling a number of smaller datasets to form a larger dataset, understanding the underlying variability between different acquisition and processing protocols across the aggregated dataset (termed “batch effects”) is critical. The presence of variation in the training dataset is important as it more closely reflects the true underlying data distribution and, thus, may enhance the overall generalizability of the tool. However, the impact of batch effects must be carefully evaluated in order to avoid undesirable effects that, for example, may reduce performance measures. Batch effects can result from many sources, including differences in acquisition equipment, imaging technique and parameters, as well as applied processing methodologies. Their impact, both beneficial and adversarial, must be considered when developing tools to ensure that their outputs are related to the proposed clinical or research question (*i.e*., actual disease-related or pathological changes) and are not simply due to the peculiarities of underlying batch effects in the aggregated dataset. We reviewed applications of DL in structural brain MR imaging that aggregated images from neuroimaging datasets, typically acquired at multiple sites. We examined datasets containing both healthy control participants and patients that were acquired using varying acquisition protocols. First, we discussed issues around *Data Access* and enumerated the key characteristics of some commonly used publicly available brain datasets. Then we reviewed methods for correcting batch effects by exploring the two main classes of approaches: *Data Harmonization* that uses data standardization, quality control protocols or other similar algorithms and procedures to *explicitly* understand and minimize unwanted batch effects; and *Domain Adaptation* that develops DL tools that *implicitly* handle the batch effects by using approaches to achieve reliable and robust results. In this narrative review, we highlighted the advantages and disadvantages of both classes of DL approaches, and described key challenges to be addressed in future studies.

## 1. Introduction

Structural magnetic resonance (MR) imaging is a critical component in many clinical and research brain studies (Chalavi et al., 2012; Kim et al., 2014; Fillmore et al., 2015). These and similar studies allow for the analysis and quantification of brain tissues and important brain structures (Cover et al., 2017; Moeskops et al., 2018; Smith et al., 2019), and for the accurate detection of brain pathology (or abnormalities) (Sandeep et al., 2006; Kim et al., 2014; Lian et al., 2021). Improvements in MR acquisition techniques over the past few years have allowed for the detection of increasingly smaller structures and more subtle abnormalities. However, these improvements also have led to an exponential increase in the volume and complexity of the image data to be processed and analyzed. The contemporaneous growth in the number and size of multi-site MR imaging studies has resulted in additional challenges related to managing large data volumes and understanding the increased imaging variability, particularly when aggregating data acquired at different research facilities or with different acquisition protocols (Nieuwenhuis et al., 2017).

The ability to appropriately manage, understand and aggregate large, multi-site and heterogeneous datasets is a key requirement when developing automated, computer-assisted tools to study brain structures and monitor brain abnormalities (Leite et al., 2016; Pinheiro et al., 2019; Gauriau et al., 2021). This requirement is particularly true when developing deep learning (DL)-based methods (Lundervold and Lundervold, 2019; Pan et al., 2020). When experiments are performed with smaller, more homogeneous datasets, the tool performance is often limited to the specific, tightly prescribed, cohort under study (Maier et al., 2018; Saba et al., 2019). The experimental results demonstrated in larger, more heterogeneous datasets are typically found to be more reliable and generalizable, allowing tool performance to be extrapolated to other cohorts (Tofts and Collins, 2011; Chalavi et al., 2012). However, images acquired at different sites can present varying characteristics due to differences in acquisition parameters, site procedures, and scanner configuration. These effects represent scan variability and commonly are termed batch effects. If not well understood and appropriately addressed, batch effects may lead to misleading or unreliable results in many applications (Pizarro et al., 2019).

The challenges of batch effects may be further exacerbated in the era of DL as new, large, multi-site, heterogeneous datasets are created by combining (or aggregating) multiple previously acquired datasets (Bento et al., 2019; Lee et al., 2020; Zlochower et al., 2020). The impact of data variability must be first understood to ensure that these models are answering the proposed research question, not only in the presence of inherent image variability, but also after addressing potential misclassification or misdiagnosis attributable to this variability (Obuchowicz et al., 2020). Large and heterogeneous datasets are key requirements for model development to avoid limiting the model to a specific cohort. This observation is especially true for models that require large and heterogeneous datasets during the development phase in order to ensure model convergence and avoid overfitting (Maier et al., 2018).

Generalizable DL is an important objective when developing tools using MR brain images (Kuijf et al., 2019). Tools need to assure that their results are reliable, even when broadly applied (*i.e*., they work with data other than that used for training). The improved performance of DL tools designed with heterogeneous datasets allows for superior benchmarking, primarily by avoiding the use of handcrafted features that are more disease- or image-cohort specific (Shen et al., 2017). Generalized DL models that, for example, successfully process multi-site, heterogeneous data acquired on different scanners, are more likely to be adopted in broader clinical and medical research environments (Akkus et al., 2017).

While our focus here is on brain imaging, many of these concepts are general and, broadly speaking, applicable to imaging studies of other anatomical regions. The study and development of DL models with large heterogeneous data are relevant to a variety of applications, and also, for ensuring imaging quality control and protocol compliance (Currie et al., 2019). Improving the reliability, generalization, and interpretability of these models would enhance application in multiple research areas (Jiang et al., 2018) and, importantly, accelerate translation to the clinic (Faria et al., 2015). These expectations are in particularly true for data acquired using different parameters or at different sites, longitudinal studies in which the scan protocol may change over time, assessment of different disease processes, or different progression rates in the same disease.

**Figure 1:**
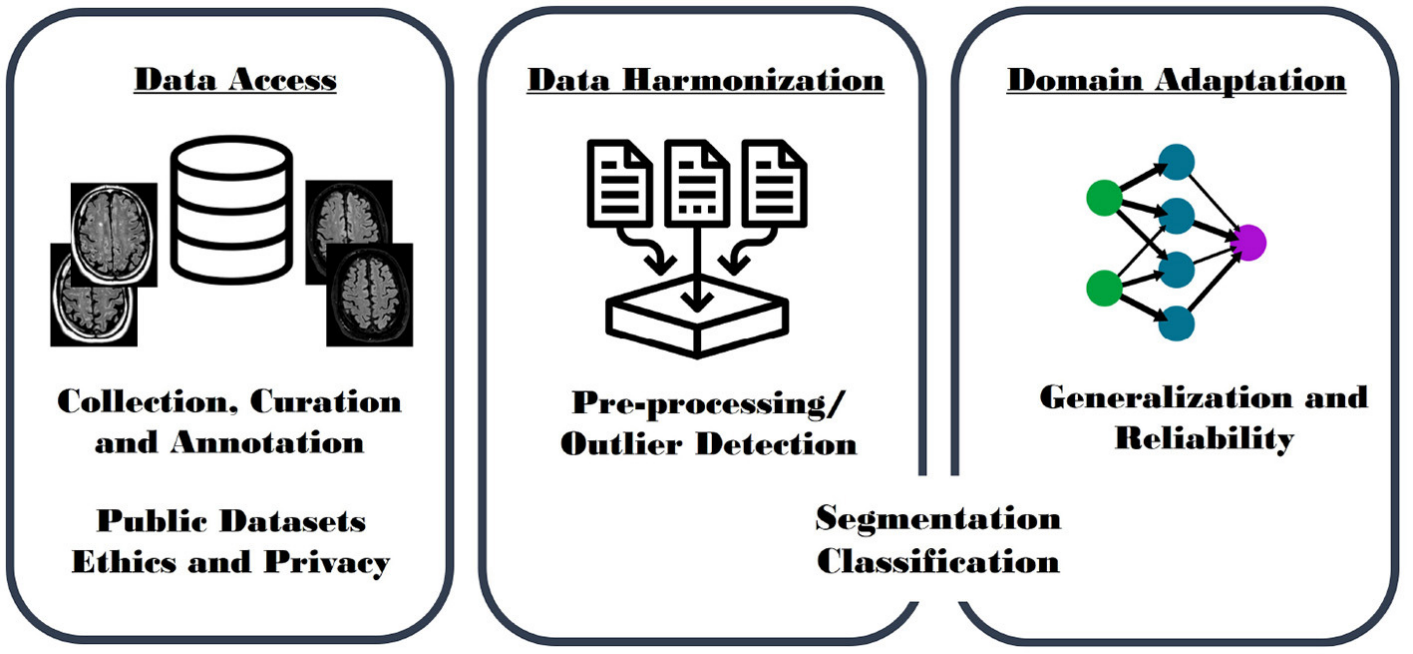
Graphical paper outline. *Data Access* (left): including description of heterogeneous datasets due to variations in acquisition, curation, and annotation; challenges with access to publicly available data, and issues relating to ethics and privacy concerns. *Data Harmonization* (middle): including model development from standardized image sets generated by performing pre-processing and outlier detection algorithms. *Domain Adaptation* (right): including use of advanced techniques and models that improve generalization and reliability by using techniques, such as domain transfer and multi-task learning, as well as adversarial network approaches. Data Harmonization and Domain Adaptation are further organized by proposed task: segmentation and classification models.

Our goal was to investigate issues relating to batch effects when developing DL models, and potential future application of DL tools that study, segment, classify and quantify brain aging, and brain abnormalities. We have focused this review on development, evaluation, and the specific challenges of conducting large, multi-site, heterogeneous studies. Such studies attempt to leverage the power of data collected at different sites. We concentrate on three main aspects (Figure 1): (A) *Data Access* (section 2) with a focus on data collection, specifically on public datasets; (B) *Data Harmonization* (section 3) including works aiming to explicitly standardize (or harmonize) heterogeneous MR imaging datasets by, for example, performing pre-processing and outlier detection methods; and (C) *Domain Adaptation* (section 4) including surveying models implicitly optimized to present generalizable and reliable results on heterogeneous datasets.

More conventional machine learning methods have studied batch effects in heterogenous, multi-center, MR head imaging datasets. Bento et al. (2021), for example, demonstrated accuracy rates >98% for a model determining key image acquisition parameters, such as MR vendor and magnetic field strength. Data harmonization approaches aim to minimize the impact of these and other acquisition variables by making it more difficult to distinguish images acquired at different sites using different acquisition parameters. Data harmonization methods attempt to explicitly minimize the influence of acquisition variation. Domain adaptation approaches, in contrast, seek to implicitly account for batch effects by accounting for this variability within the proposed model. These methods usually require the creation of a domain-diverse dataset to train the models, ultimately increasing the generalizability, minimizing the divergence criterion between the data distributions, and facilitating domain-independent decision making. To ensure clarity, the review of data harmonization and domain adaptation was further organized by proposed image processing task: segmentation or classification (Figure 1).

In this narrative review, we identified key papers describing DL models applied to adult human structural brain MR imaging problems, especially those processing commonly acquired structural MR sequences like T1-weighted (T1-w), T2-weighted (T2-w), and fluid-attenuated inversion recovery (FLAIR) imaging. In our non-exhaustive search on PubMed and of conference proceedings, we used “deep learning” and “heterogeneous brain MR imaging” as principal keywords. Additional keywords, including “multi-center MR dataset,” “trustworthy AI,” “data bias,” “fairness,” “robustification,” “invariant,” “out-of-distribution sample,” “domain adaptation,” and “domain shift” were used to refine the search for our review. We included only English-language reports published prior to 31 August 2021. We noted the significant use of technical jargon in this area of research, making literature review and benchmarking challenging. The intent of this review was to comprehensively and critically overview the use of large, multi-site, heterogeneous brain imaging dataset in DL. We highlighted and provided representative examples of significant areas of research activity, identified key challenges and gaps in the research literature, and proposed important future needs.

## 2. Data Access

Large, heterogeneous datasets are required to develop solutions for many DL problems in adult brain imaging. Typically, these datasets contain images acquired on scanners from multiple sites and may include acquisitions on scanners from different vendors operating at different magnetic field strengths, using similar but not necessarily the same acquisition parameters (Guio et al., 2016). In this section, we review the benefits and challenges of accessing heterogeneous data, as well as explore difficulties when using public datasets, as well as emerging ethical and privacy concerns related to the usage of medical imaging data.

**Figure 2:**
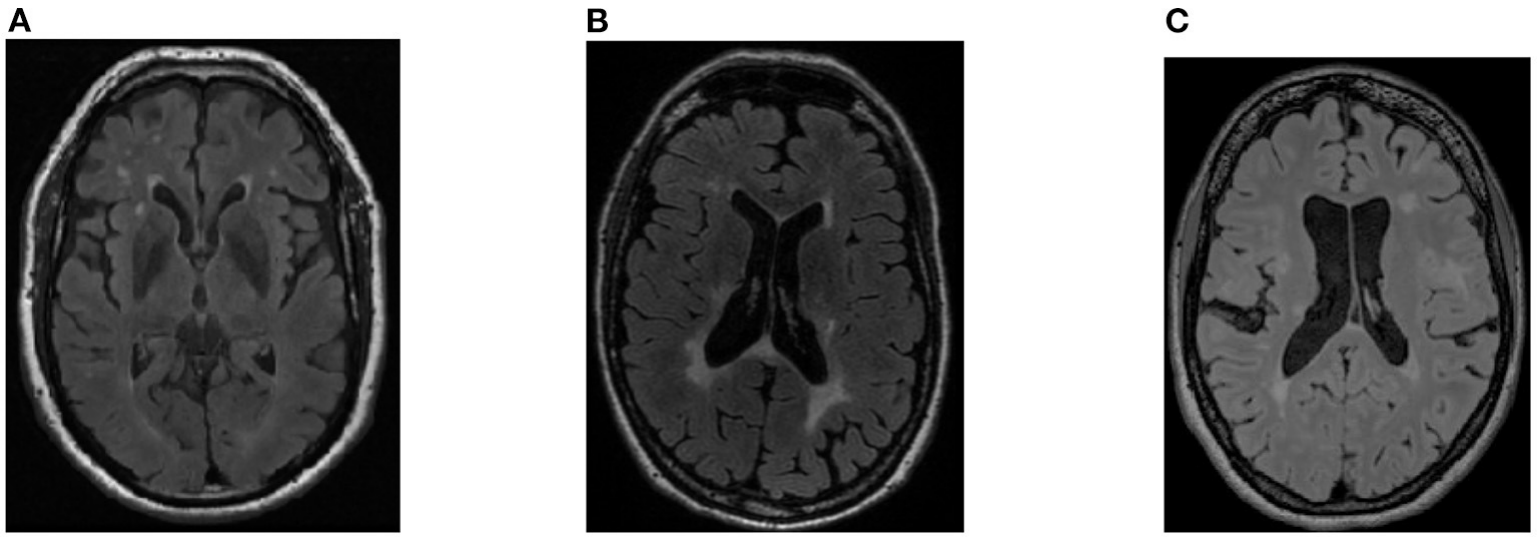
Example of fluid-attenuated inversion recovery (FLAIR) images in a multi-site dataset: **(A)** healthy control participant imaged on a specific scanner vendor; **(B)** a patient with a pathology (white matter hyperintensities, WMH, Wardlaw et al., 2013) imaged on the same scanner vendor; **(C)** a second patient with WMH pathology imaged on a different scanner vendor. Images acquired using different scanner vendors may present different image contrasts, shape, and other characteristics. These varying characteristics may impact the DL models development and performance due to unwanted correlations in the dataset (that are not correlated with the pathology occurrence).

Training of DL models benefits from using large datasets that sufficiently represent the qualities of the population under study. The dataset should not only represent the phenomena under investigation (such as presence of a specific pathology) but also include examples of individuals with confounding phenomena and without the phenomena, as well as incorporating the range of anticipated batch effects. These effects describe variation due to differences in data acquisition, processing, curation, or annotation steps. Appropriate incorporation of this variability is thought to be essential to model generalization (Balachandar et al., 2020). One way of achieving this goal is to combine data from different datasets; an approach that facilitates the conduct of experiments on a larger and more diverse set of samples. Attention, however, must be paid to prevent introduction of unwanted correlations in the aggregated dataset (*e.g*., having individuals with pathology imaged on a scanner from one vendor and individuals without pathology scanned on another vendor) (Figure 2). Often, publicly accessible, open-access repositories are a vital source of the data needed for training, validating, and testing DL algorithms, specially when benchmarking algorithms (Prior et al., 2020).

Compared to other DL applications, specifically non-medical applications, many brain imaging datasets present with a limited amount of “normal” data. Such data are obtained from presumed healthy, normal, or control participants and are frequently limited in number and diversity, typically because of either the cost of conducting an MR imaging procedure or challenges in accessing an MR scanner by non-patients (Larrazabal et al., 2020). When developing DL models, the lack of normal data presents a challenge, raising the requirement for larger datasets for training using strategies like data augmentation and combination of different datasets, or using more advanced DL strategies like transfer learning and domain adaptation (Teh, 2020).

Another significant obstacle in developing and implementing supervised DL models is the often limited availability of appropriately annotated images in these datasets. Manual labeling by experts commonly is used to support supervised learning or to assess model performance. Some datasets present labels that can support supervised learning strategies for classification tasks (often related to the patient clinical diagnosis), or segmentation tasks (brain structures or brain abnormalities related to a specific pathology). Manual labeling, however, can be time-consuming, expensive, prone to errors, and may present with unacceptable intra- and inter-expert variation (Obuchowicz et al., 2020). There are different approaches to overcome these challenges, such as using consensus methods, like simultaneous truth and performance level estimation (STAPLE, Warfield et al., 2004), and unsupervised approaches (Shen, 2013; Souza et al., 2018).

Finally, to fully develop reliable, validated, and reproducible research, it is also necessary to ensure quality of the acquired data. There are many factors that can impact acquisition quality. For instance, long acquisition times can adversely affect patient comfort, increasing the possibility of image artifact due to motion (Fantini et al., 2021). For this reason, an image quality control step is a prerequisite in large projects, specially when involving multiple sites. Quality control ensure minimum requirements and quality standards for the medical images to be use in supporting diagnosis and treatment responses (Kim et al., 2019).

There are many publicly available brain MR datasets. These datasets can include presumed healthy control (HC) participants, as well as patients diagnosed with different pathologies like Alzheimer's disease (AD) or mild cognitive impairment (MCI), autism, brain cancer (*e.g*., glioblastoma multiforme, GBM), multiple sclerosis (MS), and Parkinson's disease. Most of these datasets include adults from around 18 years of age through to 80 years of age and older, including both male and female participants.

Many datasets present a single MR modality (*i.e*., T1-w volume images), while others support multi-modality studies. We summarized commonly used public brain MR imaging datasets (having >30 participants, Table 1). Of note, several of these databases have been made available during conferences like the Medical Image Computing and Computer Assisted Intervention (MICCAI) Society. In our tabular summary, we have included the public datasets that were used in many of the studies described in the following sections. We purposefully did not include subscription or pay-to-access brain imaging datasets.

**Table 1:** Summary of some publicly available MR brain imaging datasets.

	**#**	**Acquisition**	**Longitudinal**	**Sample**	**Availability**
**Dataset**	**Participants**	**sequences**	**data**	**population**	**of labels**
ABIDE (Di Martino et al., 2014)	1112	T1-w	NO	HC, Autism	YES
ADNI (Wyman et al., 2013)	2542	T1-w, T2-w, FLAIR, DTI	YES	HC, MCI, AD	NO
Calgary-Campinas-359 (CC-359) (Souza et al., 2017)	359	T1-w	NO	HC	NO
Cambridge Center for Aging Neuroscience (Taylor et al., 2017)	653	T1-w, T2-w	NO	HC	YES
Connectome Coordination Facility (CCF) - HCP Young Adult					
https://www.humanconnectome.org/study/hcp-young-adult	1113	T1-w	NO	HC	YES
Dallas Lifespan Brain Study (Bischof and Park, 2015)	315	T1-w	NO	HC	YES
Information eXtraction from Images (IXI)					
(brain-development.org/ixi-dataset/)	600	T1-w, T2-w, PD, DWI	NO	HC	NO
MICCAI 2015 BRATS (Menze et al., 2015)	262	T1-w, T2-w, FLAIR	NO	GBM	YES
MICCAI 2019 WMH Segmentation (Kuijf et al., 2019)	60	T1-w, FLAIR	NO	SVD	YES
MIRIAD (Malone et al., 2013)	69	T1-w	YES	HC, AD	YES
MR-MS (Lesjak et al., 2018)	36	T1-w, T2-w, FLAIR	YES	MS	YES
Neuroimaging Tools and Resources Collaboratory (NITRC)					
(https://www.nitrc.org/)	65	T1-w, T2-w, PD	NO	HC	YES
OASIS (Fillmore et al., 2015)	1664	T1-w	NO	AD	NO
PPMI (Simuni et al., 2016)	1460	T1-w	YES	HC, Prodomal Parkinson's	YES
Southwest University Adult Lifespan Dataset (Wei et al., 2018)	494	T1-w	NO	HC	YES
TCIA (public.cancerimagingarchive.net/nbia-search/)	955	T1-w	NO	GBM	YES

*The list includes relevant information, such as number of participants, acquisition sequences, if longitudinal data are available, the sampled population and if manual labels are available. AD, Alzheimer's disease; DWI, diffusion-weighted imaging; DTI , diffusion tensor imaging; FLAIR, fluid-attenuated inversion recovery; GBM, glioblastoma; HC, healthy control; MCI, mild cognitive impairment; MS, multiple sclerosis; PD, proton density; SVD, small vessel disease; T1-w, T1-weighted; T2-w, T2-weighted*.

Pooling of datasets is common, despite the complexities of effectively combining datasets (*i.e*., dataset aggregation). Researchers often express disappointment with having to browse disconnected data repositories with non-intuitive interfaces in order to access and select data. They must also deal with varied levels of data quality and data completeness, requiring additional time and resources to prepare data for their analysis. In addition, once data are obtained, the connection to the source archive is often lost making changes difficult to track, potentially leading to inconsistencies in the data. Some support tools are available, such as Datalad (https://www.datalad.org/), which provide a versioning system for shared datasets. However, personnel with specialized training are required to run these tools. There are also newer initiatives, such as the Connectome Coordination Facility (https://www.humanconnectome.org/), which attempts to overcome some of these issues by adopting an integrated focus on data acquisition and data infrastructure to both host and distribute data, and data harmonization to assure that data acquired at multiple institutions across different studies are as comparable possible.

Accessing large, heterogeneous datasets can also raise a number of ethical and privacy concerns. Studies involving human participants require ethical approval from one or more local Research Ethics Boards or equivalent. Normally, approval is granted only after providing information related to (1) the procedures for participant recruitment, (2) the specific process of obtaining informed consent, (3) the nature of the material that will be collected, and (4) the uses, both primary and secondary, of the acquired data (Larson et al., 2020). These considerations are pertinent topics for discussion when working with medical images, since online storage of digital images facilities data access (Lotan et al., 2020). Critically, medical images differ from other health data types in a few important aspects. Electronic health records, for example, are comparatively easy to aggregate and de-identify (or anonymize). Medical images, in comparison, require further, more detailed consideration to ensure appropriate data handling and protection of individual privacy (Balthazar et al., 2018; Larson and Boland, 2019).

Potentially identifiable, clinical data must be de-identified and carefully safeguarded. Data sharing and aggregation for research and development should only be allowed when those with data access are identified and agree to respect the ethics and privacy regulations (Larson et al., 2020).

## 3. Data Harmonization

**Figure 3:**
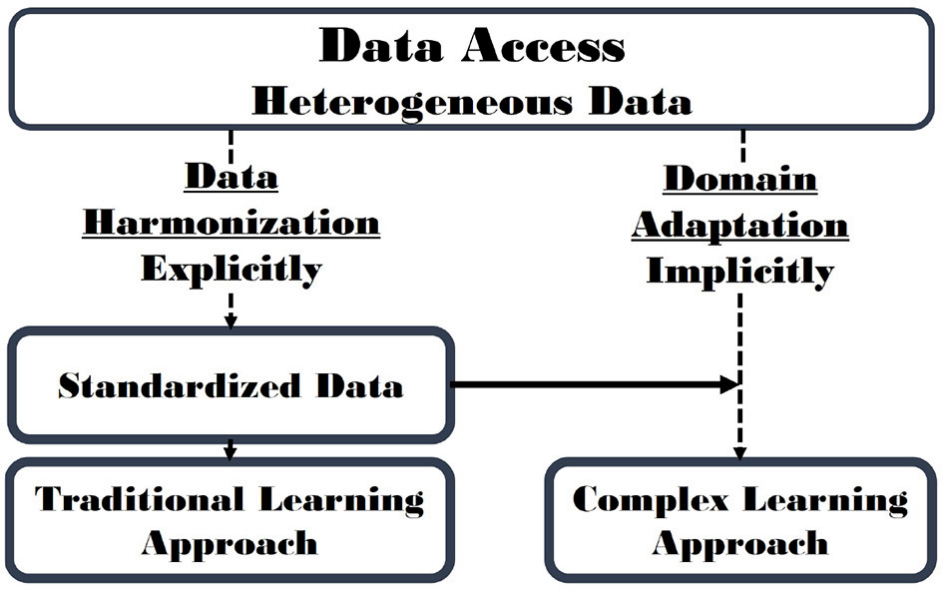
Data Harmonization (section 3) and Domain Adaptation (section 4) are related concepts that address issues related to batch effect correction. Data Harmonization attempts to standardize images, explicitly minimizing the batch effects. Conversely, Domain Adaptation implicitly corrects batch effects while achieving the modeling objective. A hybrid approach that first includes elements of data harmonization and is followed by a domain adaptation approach (solid horizontal arrow) is possible and may lead to improved results.

With the proliferation of multi-site neuroimaging studies, there is an emerging need to more appropriately handle the non-pathological batch effects, particularly after dataset aggregation. If improperly addressed, these effects can potentially hinder the detection of imaging features associated with the clinical covariates of interest (*i.e*., imaging evidence of disease-related pathology) and, worse, may cause spurious or erroneous findings. Figure 3 describes an organizational structure for categorizing methods for processing heterogeneous data. In this section, methods that use data harmonization approaches will be reviewed and in section 4 methods that use Domain Adaptation approaches will be described. Table 2 summarizes key data management strategies by batch effect correction approach (data harmonization *vs*. domain adaptation) and principle task (segmentation *vs*. classification).

**Table 2:** Summary of reviewed papers organized by batch effect correction approach (denoted by shading): explicitly using data harmonization *vs*. implicitly using domain adaptation; and principal task (segmentation vs. classification).

	**Batch effect**	**Principle**	**Participant**	**Main**
**Publication**	**correction approach**	**task**	**population**	**aim**
Smith et al. (2019)	Explicit	N/A	SVD	Propose a harmonizing framework for neuroimaging studies
Jovicich et al. (2006)	Explicit	N/A	Phantom	Improve reproducibility in morphometric studies
Fortin et al. (2018)	Explicit	N/A	HC	Investigate scanner effects on cortical thickness measurements
Li et al. (2020)	Explicit	N/A	HC	Propose a denoising method to reduce variance related to scanner
Zeng et al. (2018)	Explicit	N/A	Synthetic data and HC	Perform super-resolution reconstructions
Gauriau et al. (2021)	Explicit	Segmentation	Various pathologies	Study abnormalities in MR images related to the presence of brain pathology
Despotovic et al. (2015)	Explicit	Segmentation	HC	Compare different brain segmentation strategies with focus on the pre-processing
Liao et al. (2008)	Explicit	Segmentation	Phantoms and HC	Study the impact of intensity inhomogeneities or bias fields
Ahmed et al. (1999)	Explicit	Segmentation	HC	Estimate and compensate for intensity inhomogeneities
Shinohara et al. (2017)	Explicit	Segmentation	MS	Propose several automated lesion-detection and whole-brain analysis using protocol harmonization
Sajja et al. (2006)	Explicit	Segmentation	MS	Minimize false-positive lesion classification on brain segmentation
Dewey et al. (2019)	Explicit	Segmentation	HC	Propose a data harmonization-segmentation method based on image contrast (DeepHarmony)
Swati et al. (2019)	Explicit	Classification	GBM and HC	Perform brain tumor classification using transfer learning considering unbalance training data
Van Leemput et al. (1999)	Explicit	Classification	MS	Propose a fully automated bias field correction prior to tissue classification
Tohka et al. (2016)	Explicit	Classification	AD, MCI and HC	Compare different feature selection approaches in dementia classification
Sundaresan et al. (2021b)	Implicit	Segmentation	AD, MS, Stroke and SVD	Develop domain adaptation strategies to segment brain lesions
Zeng et al. (2020)	Implicit	Segmentation		Compare lesion segmentation methods according to improve translation to clinical environment
Orbes-Arteaga et al. (2019)	Implicit	Segmentation	SVD	Propose a domain adaptation strategy for using data augmentation and adversarial networks
Ghafoorian et al. (2017)	Implicit	Segmentation	SVD	Study how to properly apply domain adaptation: required amount of data from new domain and portion of model to be retrained
Akkus et al. (2017)	Implicit	Segmentation		Survey brain MR imaging segmentation methods according to the usage of data augmentation and transfer learning
Karani et al. (2018)	Implicit	Segmentation	HC	Propose a brain structure segmentation method for single CNN with shared convolutional filters and domain-specific batch normalization layers
Ackaouy et al. (2020)	Implicit	Segmentation	GBM and HC	Describe an unsupervised domain-shift approach for brain abnormality segmentation
Kondrateva et al. (2021)	Implicit	Classification		Analyze and compare different approaches for domain-shift problem with focus on advanced data processing, auto-encoding neural networks and their domain-invariant variations, model architecture enhancing, and feature training
Hofer et al. (2017)	Implicit	Classification	AD and HC	Propose an approach to perform adaptation in feature space directly to reduce domain shift impact
Islam and Zhang (2018)	Implicit	Classification	AD and HC	Identify different stages of AD and obtained superior performance for diagnosing early-stage disease using ensemble Deep CNNs
Jain et al. (2019)	Implicit	Classification	AD and HC	Propose AD classification approach based on transfer learning minimizing the pre-processing steps
Zhang et al. (2019)	Implicit	Classification	AD and HC	Perform brain disease identification using unsupervised conditional consensus adversarial network

*The study participant population and main aim are provided. AD, Alzheimer's disease; GBM, glioblastoma; HC, healthy control; MCI, mild cognitive impairment; MS, multiple sclerosis; N/A, Not-applicable; SVD, small vessel disease*.

An important approach when developing DL models using large and heterogeneous datasets is harmonization and/or standardization. Efforts to achieve data harmonization of heterogeneous datasets (Onofrey et al., 2019) include minimizing variability by removing poor quality samples (*i.e*., outlier detection Bento et al., 2018) and by applying procedures for image standardization (*i.e*., pre-processing Kidoh et al., 2020). The use of content-based medical image retrieval methods are also relevant to data harmonization as they apply techniques for retrieving cohorts of similar images from a larger image database, (Liu et al., 2017). Outlier detection, pre-processing and content-based retrieval methods can decrease the sources of unwanted variability in image datasets. These techniques that modify images may result in the introduction of undesired changes (*e.g*., image artifacts) or may result in the loss of subtle biomarkers and abnormalities, particularly if image smoothing techniques are used (McVeigh et al., 1985).

Smith et al. (2019) identified gaps in our knowledge and proposed to develop tools for harmonizing imaging and analysis by proposing a framework for neuroimaging biomarker development that was based on (1) validating repeatability and reproducibility, (2) attention to biological (or clinical) principles, and (3) assessment of the feasibility of implementation. Their platform (www.harness-neuroimaging.org) comprises an MR imaging repository with specific acquisition protocols, software database, rating scales, and case report forms that is suitable for cerebral small vessel disease applications. Similar harmonizing frameworks would be generalizable to other brain disorders.

The effects of MR scanner gradient non-linearity have on the reproducibility of multi-site human MR imaging is one example of a situation that depends on data harmonization. Gradient non-linearity was investigated in order to facilitate precise, quantitative, platform-independent, multi-site evaluation by Jovicich et al. (2006) who applied an image distortion correction method based on spherical harmonic description of the gradient errors. They verified the method using phantom data and then applied it to the brain image data from a group of participants scanned twice at multiple sites using different 1.5 T MR scanners. Imaging variability within sites and in multi-site studies was assessed by evaluating the reproducibility of voxel-based image intensities. Reproducibility was improved after gradient non-linearity distortion correction, suggesting that this data harmonization scheme may improve reproducibility in morphometric studies. The correction for gradient non-linearity errors has the potential for improving the accuracy of morphometric analysis in longitudinal and multi-site imaging studies, by improving both geometric accuracy and image intensity reproducibility. These corrections, while beneficial in multi-site studies, however, do not account for all the sources of image intensity variability.

Fortin et al. (2018) also investigated MR scanner effects in large multi-site studies, specifically on cortical thickness measurements using eleven MR scanners. They proposed a set of tools (based on earlier work in gene expression Johnson et al., 2007) aiming to identify scanner effects that are generalizable to other modalities. Authors showed that the proposed approach not only minimize batch effects, but also increased reproducibility, based on the presented statistical analyses by mitigating batch effects (Fortin et al., 2018). In another study, Li et al. (2020) proposed to identify and remove effects related to the scanner from multi-site MR imaging. They proposed a data-driven approach that applied independent component analysis (ICA) to test the proposed method, developed on a single 3 T scanner. Their proposed denoising method showed a reduction of variance related to scanner. The method showed promising findings for minimizing batch effects in heterogeneous and multi-site studies containing data acquired in different scanners.

A deep convolutional neural network model was proposed Zeng et al. (2018), aiming to perform single- and multi-contrast super-resolution reconstructions. Their results, using synthetic and real brain MR imaging data, showed that the proposed model outperforms state-of-the-art MR imaging super-resolution methods, considering visual quality and quantitative measurements such as peak signal-to-noise ratio (pSNR) and structural similarity index (SSIM). This improved performance was obtained when the training and test images were from the same dataset, while the super-resolution results for the multi-site data were poor. These findings indicated that these training and testing images have large variations resulting from the use of different datasets. For this reason, the authors suggested the training and testing images should come from the same dataset, having data acquired under the same experimental conditions. Another limitation was related to the size of the dataset, a large dataset was required to train their proposed model. Training of their models was also a time-consuming task.

Another DL approach to perform data classification was proposed by Gauriau et al. (2021). The goal of their study was to distinguish MR images for the presence of brain pathology (*i.e*., either “likely normal” or “likely abnormal”) using FLAIR images. The experimental dataset comprised over 10,000 patients with a broad variety of pathologic conditions, including neoplasms, hemorrhages, and infarcts. They performed a pre-processing step that resized and normalized the images, before performing experiments across datasets to evaluate model generalization. The model showed good performance in differentiating “likely normal” from “likely abnormal” brain examinations using multi-site data. This study also highlighted that some pathologic conditions with subtle imaging findings were not readily visible on FLAIR images, illustrating a possible limitation of the model. They proposed to mitigate this limitation by using data acquired from multiple MR sequence acquisitions (resulting in different image contrasts) in future studies.

The key advantage of harmonization approaches is that they attempt to further understand data variability before attempting to reduce it. Thus, they provide an implicit method to improve image curation and overall dataset quality. They also allow the subsequent application of less complex learning models that focus only on disease-related pathology. The use of less complex models can potentially improve model interpretability (Figure 3). However, the steps and techniques commonly used to standardize data are tailored to specific harmonization of datasets. When new data is input into the analysis, this data much be first re-evaluated and the model possibly retrained, to ensure that appropriate harmonization approaches are used.

### 3.1. Segmentation

Image segmentation is an important brain MR image processing task that is commonly used for visualizing and measuring anatomical structures. It is a necessary step for brain morphometric, for delineating pathological regions, and for surgical planning and image-guided interventions (Lorenzo et al., 2019). In the last few decades, a series of brain segmentation techniques of varying accuracy and degrees of complexity have been developed and reported in the literature. Undeniable, computerized image-segmentation methods have shown much potential and application in computer-aided diagnosis and treatment planning. In this section, we focus on the role of data harmonization by studying data variability applied to human brain segmentation from MR images.

Despotovic et al. (2015) compared different approaches for brain segmentation, summarizing their characteristics, advantages and disadvantages. Special focus was given to the MR imaging pre-processing step, including image registration, bias field correction, and removal of non-brain tissues. The authors showed that the selection of the most appropriate technique for a given application is a difficult task, since new segmentation problems emerge and new solutions are continuously proposed. In fact, Despotovic et al. highlighted that in many cases, a combination of several techniques may be a requirement to achieve a reliable segmentation. Another important concept in brain segmentation is the integration of multi-modal information (*i.e*., images acquired from different modalities or at varying points in time).

The impact of intensity inhomogeneities or bias fields in MR images have also been examined. Liao et al. (2008) presented work on a fast, spatially constrained kernel clustering algorithm for segmenting medical brain MR, specially correcting bias fields in MR imaging data. Their algorithm uses a kernel technique to map image data to higher dimensional kernel space, improving the separability of data and providing greater potential for effectively segmenting MR imaging data. They also proposed an approach for correcting spurious intensity variation in MR images. The fast kernel clustering and bias field correction are beneficial, and when used in an iterative manner have dramatically reduced the complexity of kernel clustering. Experiments on both phantoms and real MR images showed that the proposed algorithm generally outperformed the corresponding traditional algorithms when segmenting MR imaging data corrupted by high noise and bias fields.

Ahmed et al. (1999) proposed a brain segmentation that focuses on data harmonization by estimating intensity inhomogeneities for segmentation of MR imaging data. Their algorithm modifies the objective function of the standard fuzzy c-means algorithm to compensate for such inhomogeneities. Their proposal allows a pixel labeling to be influence by the labels in its immediate neighborhood, acting as a regularizer, leading to a more homogeneous labeling results. Experiments performed on both synthetic data and MR images demonstrated the effectiveness and efficiency of the proposed algorithm.

A different approach to segmenting lesions in MS patients from T1- and T2-weighted MR images was described by Shinohara et al. (2017). This paper address an important topic that is multi-site variability by imaging a volunteer with relapsing-remitting MS twice (scan-rescan testing) at seven sites associated with the North American Imaging in MS Cooperative Steering Committee. This committee developed a uniform high-resolution 3 T MR imaging protocol for quantifying cerebral lesions and atrophy and implemented it at the sites. Expert image segmentation maps were manually obtained for lesions on both the T1-w and T2-w images to allow the development of supervised techniques. Several automated lesion-detection and whole-brain, cortical, and deep gray matter segmentation pipelines were assessed, and statistical analyses performed to assess variability as well as systematic biases in the volume measurements across sites. The study found that even in multi-site studies with consistent scanner field strength and vendor after protocol harmonization, systematic differences can lead to severe biases in volumetric analyses.

Sajja et al. (2006) tackled another common issue when segmenting MR images: false-positive lesion classification on segmented brain MR images, which can be a major problem when determining lesion volumes in MS and other patients. Their approach used proton density (PD)-weighted, T2-w and FLAIR images and involved lesion classification using the Parzen window classifier (Jain and Ramaswami, 1988). The performance of this algorithm was evaluated on 23 MS patients. Contextual information was exploited to minimizing the false-negative lesion classifications using a hidden Markov random field-expectation maximization (HMRF-EM) algorithm and lesions were delineated using fuzzy connectivity. One of the main advantages of the proposed approach is the translation to other brain pathologies with minor modifications. The proposed method, however, does have a limitation related to lesion overestimation, mainly impacting smaller lesion loads. Another limitation was related to the need of human intervention.

The final example study in the data harmonization-segmentation subsection proposes a method of contrast harmonization, called DeepHarmony. This method uses a U-net-based DL architecture to produce images with consistent contrast (Dewey et al., 2019). Images harmonized with DeepHarmony have shown significant improvement in consistency of volume quantification between scanning protocols. To provide training data, a small cohort (*n* = 8) was scanned using two different protocols (overlapping participant cohort). Contrast harmonization showed that the atrophy estimate were affected by protocol change, impacting the DeepHarmony findings. The data from overlapping participant cohort allows overcoming inconsistencies in segmentation caused by the batch effects. This study of batch effects allow the design of long-term studies, without invalidation of results previously acquired. Further, the authors highlighted some limitations for longitudinal studies, which could be overcome by augmenting the DeepHarmony methodology with improvements such as fully 3D networks or adversarial training, which have been shown to improve accuracy and perceptual quality in computer vision and medical imaging tasks.

Even when essentially all acquisition protocols parameters are harmonized, combining studies still can present variability due to small, unaccounted for differences between scanners. In most cases, specific harmonization techniques are tailored to standardize specific combinations of datasets. Thus, data harmonization in image segmentation is an area that must be constantly evolve to allow the aggregation of larger datasets with more sources of data variability.

### 3.2. Classification

In this section, important studies that perform data harmonization prior to image classification are reviewed. Image classification overcomes the semantic gap between the low-level visual information captured in the MR image and the high-level information (descriptor) perceived by a human evaluator. Traditional machine learning classification techniques focus only on low-level or high-level features, some use some hand-crafted features to reduce the semantic gap, and most require the combination of good feature extraction and classification methods. DL has recently shown great progress and, specially, deep convolution neural networks (CNNs) have shown much success in challenging image classification tasks. DL is very powerful in that feature representation can effectively depict both low-level and high-level information.

For most medical imaging applications, the training datasets are small, therefore, it is often a challenging task to apply DL and train models from scratch with only access to small datasets. To overcome the limitation of small training dataset sizes, different net approaches have been examined. For example, a brain tumor classification using a pre-trained deep CNN model based on transfer learning (Swati et al., 2019). Experiments were performed using T1-w MR imaging data. The proposed approach did not use handcrafted features and required less pre-processing. The results were compared not only with traditional classification models but also to other CNN approaches, outperforming state-of-the-art classifiers. This work demonstrated the learning transferability from natural images to medical brain MR imaging, by using a transfer learning strategy as feature extractor (without additional domain-specific training) that was trained separately from the classifier training. One limitation was the in ability to perform fine-tuning experiments using volumetric images because of the size of the dataset.

Van Leemput et al. (1999) evaluated the usage of a fully automated bias field correction prior to tissue classification. In this paper, the MR signal was modeled as a random process with a parametric probability distribution with inhomogeneity field corruption. The proposed method aims to improve the likelihood of model parameters using an iterative strategy, interleaving pixel classification with the estimation of class distribution and bias field parameters. The proposed approach requires no user interaction, allowing objective and reproducible results. Experiments were performed on simulated data and on various MR datasets, to illustrate performance on various MR images with field inhomogeneities, and to allow the comparison to other bias correction algorithms.

In a third example, different feature selection approaches were compared for dementia classification using brain MR imaging. The objective in Tohka et al. (2016) was to separately model two classification problems using AD patients, MCI patients, and HC participants: (1) classify AD from HC, and (2) classify MCI from HC. Tohka et al. compared support vector machines (SVMs), evaluating the usage of feature selection, embedded feature selection methods, and stability selection. The use of embedded feature selection methods resulted in an optimized generalization performance for the MCI *vs*. HC classification problem. Another relevant finding was that the variability in classification accuracy due to independent samples did not typically depend on the feature selection method, and was generally found to be acceptable. A limitation of this work was that only dementia-related applications were considered. The generalization of the study findings to other brain diseases is required.

Most of the works that aim to perform brain MR classification report challenges related to low number of samples. This is one of the main reasons for the aggregating data from multiple sites, comprising both private and public datasets. Data harmonization is applied in such datasets to reduce the data variability related to the scanner, such as pre-processing algorithms, and some also require human intervention. Besides, after data harmonization, classification studies also use strategies to overcome the limited data size, such as data augmentation strategies and transfer learning. The authors suggest that the usage of 3D networks and adversarial training to tackle image variability may lead to optimize and more generalizable findings.

## 4. Domain Adaptation

Even though careful harmonization of acquisition parameters can reduce variability, inter-protocol differences become almost inevitable to arise, specially with ongoing improvements in hardware and in sequence design, even within a single-site study (McCreary et al., 2020). This observation highlights the need to develop more advanced deep-learning models that attempt to implicitly manage data variability. These advanced models are potentially less affected by the batch effects and can thus present generalizable results (Moeskops et al., 2018; Perkuhn et al., 2018). Models that implicitly account for image data variability by, for example, employing domain transfer, domain adaptation techniques, or multitask learning have been proposed (Shin et al., 2016; Wang and Deng, 2018). By analyzing and modeling image data variability, these approaches tend to provide more reliable results because their outputs are related to the underlying research question (*i.e*., pathology of interest) and are not influenced by batch effects (Cole et al., 2017). Table 2 summarizes key domain adaptation approaches by principle task (segmentation *vs*. classification).

Generative adversarial networks (GANs) are a frequently applied approach that has been successful because of its capability to generate data without explicitly modeling the probability density function and by imposing higher-order consistency on the results (Creswell et al., 2018). These results have proven to be useful in many situations including domain adaptation, data augmentation, image-to-image translation, and cross-modality translation (Xin and Babyn, 2019). The development of new methods and algorithms for the transfer of training and adaptation of domain in multi-modal medical imaging data is crucial for the development of accurate models, and their translation to the clinical practice.

It is relevant to consider the problem of domain shift in analyses of brain MR imaging data, specially in multi-site studies. Recent work in domain adaptation addresses this challenge and successfully leverages labeled data in a source domain to perform well on an unlabeled target domain.

### 4.1. Segmentation

The accuracy of CNNs may be severely degraded when segmenting images acquired on different scanners or using different protocols as compared to the training data (Dolz et al., 2018). DL-based segmentation approaches for brain MR imaging are gaining interest due to their self-learning and ability to generalize over large amounts of image data. As DL architectures mature, they are beginning to outperform previous state-of-the-art classical machine learning algorithms (Orbes-Arteaga et al., 2019). DL has also achieved great performance in non-brain fields of medical imaging (Dewey et al., 2019).

A generalizable method for brain image segmentation where data is collected from multiple scanners and sites and, therefore, affected by site/domain shifts is proposed in Aslani et al. (2020). Performance was improved by integrating an encoder-decoder network with a regularization network that included an auxiliary loss term to reduce the impact of the domain-shift problem. Experiments were evaluated on MS lesion segmentation using a private clinical dataset (117 patients from 56 different scanning sites). The proposed method was compared with other methods in the literature and showed better generalization performance in terms of Dice similarity coefficient (DSC) and positive predictive value (PPV) measures among all tested models. The authors reported that some, mainly small volume, lesions were not identified. Automatic segmentation of lesions in MR images is essential for clinical assessment, treatment planning and response to treatment follow-up in MS. Recently, the application of CNNs has increased for this task. Although these methods provide accurate segmentation, their applicability in clinical settings remains limited due to poor reproducibility across different image domains (Ackaouy et al., 2020).

Sundaresan et al. (2021a) proposed a segmentation approach for white matter hyperintensity lesions that can occur in a variety of brain diseases (including AD, MS, stroke, and small vessel disease). Experiments using domain-adaptation strategies, such as transfer learning, domain adversarial neural networks, and domain unlearning using data from three datasets. Results showed improved generalization when compared with the baseline model, an ensemble network model proposed earlier (Sundaresan et al., 2021b), demonstrating the ability of domain-adaptation techniques to learn the domain invariance between datasets.

Zeng et al. (2020) systematically reviewed different methods of lesion segmentation in MS patients. DSC and PPV metrics were used for the quantitative comparison of the methods. The authors highlighted the difficulty for model benchmarking, specially when comparing methods that used private (or proprietary) and not public datasets. Another highlight was related to the usage of DL based methods. Although such methods improved the performance of automatic segmentation methods, there is still a key challenge to directly use these methods in a clinical environment. Aggregating, using larger datasets, and potentially using publicly available data, has the potential to improve translation of DL, accelerating their application in medical imaging applications. However, there is still room for improvement of the generalizability of DL-based methods. Zeng et al. (2020) predicted that future work in MS segmentation will be a focus on domain-adaptive models from both image and feature aspects of MS segmentation.

An interesting, semi-automatic method to adapt a segmentation model from a source domain to a target domain was proposed by Orbes-Arteaga et al. (2019). The proposed algorithm method combines consistency loss with the adversarial learning. Experiments were performed on white matter hyperintensity lesion segmentation from brain MR images (using the MICCAI 2017 challenge data Kuijf et al., 2019 as the source domain and two target domains). Their method significantly outperformed other domain-adaptation methods, presenting critical findings to the translation for the clinical practice, considering scanner upgrades and multi-site trials. Their experiments also showed that the effects of data augmentation (which usually present a positive impact in the performance) failed to provide benefit in the pure adversarial setting. Orbes-Arteaga et al. showed the best performance was obtained when combining a pure adversarial setting with the proposed strategy. They highlighted one limitation in their work, related to the need for paired data.

The use of domain adaptation for white matter lesion segmentation was also proposed in Ghafoorian et al. (2017). These authors addressed two relevant topics related to transfer learning: the amount of data on the new domain that is required for a proper domain adaptation, and the portion of the pre-trained model that needs to be retrained for a specific number of samples from the new domain. During experiments, a CNN was trained using MR images of brain and the model performance of the domain-adapted network evaluated on the same task with images from a different domain. Performance was compared to using the same trained network on a new dataset, *vs*. training from scratch. The domain-adapted network outperformed training from scratch networks. When more training data becomes available, fine-tune of the shallower layers (*e.g*., the last convolutional layers) should be performed. Tuning the initial few convolutional layers that comprises domain-independent characteristics is rarely beneficial.

Akkus et al. (2017) surveyed brain MR imaging segmentation methods, describing and comparing current start-of-the-art approaches based on speed and other properties, as well as listing possible future directions in this research area. They highlighted two important findings: usage of data augmentation may minimize the requirement for larger datasets; and advocate the usage of transfer learning, sharing well-performing DL models trained on brain MR imaging (both normal and pathological samples). In brain imaging research, transfer learning has improved the generalization capability of these models in multi-site data, when compared with learning from scratch. The authors highlighted that there is still a limitation on the current state-of-the-art models related to generalization, presenting reliable results to variations in brain MR images. In practice, the DL methods performance is dependent on pre-processing, initialization, and post-processing. Training on relatively small brain imaging datasets, as compared to large-scale natural image dataset (*i.e*., ImageNet having millions of images) result in poor generalization. Akkus et al. (2017) suggested that future DL models will need to be robust to variations in brain MR imaging or have unsupervised learning capability to reduce the requirement of ground truth labels.

Karani et al. (2018) presented a study of brain structure segmentation from MR images acquired using different scanners and protocols. They addressed the generalization problem by applying multi-domain learning and treating images acquired with different scanners and protocols as samples from different, but related domains. The proposed solution is a single CNN with shared convolutional filters and domain-specific batch normalization layers, which can be tuned to new domains with only a few labeled images, using semi-supervised learning. The proposed method presented similar results to dedicated CNN trained for each scanner/protocol combination. This paper is one of the first efforts to address the limitation of usage of CNNs for medical image analysis, providing a benchmark for managing data distribution changes observed in the clinical environment.

Another paper also proposed a domain-adaptation approach but used an unsupervised domain-shift approach (Ackaouy et al., 2020). This framework, known as Seg-JDOT, used a DL model (variation of a 3D U-net architecture) to segment similarly samples from a source domain and a target domain with similar representations. A multi-site dataset (from the MICCAI 2016 challenge Commowick et al., 2018) was used to evaluate the proposed framework and showed that adaptation toward a target site can improve model performance over standard training. The authors suggest that Seg-JDOT adaptation to other neural network architectures or tasks is straightforward. However, even with effective findings in dealing with domain-adaptation problem, the inclusion of clinical sites with small number of patient may present a limitation in the results. Future studies will need to evaluate the framework with respect to other CNN architectures, different from U-net.

Although DL has achieved great performance for both brain structure and brain abnormalities segmentation tasks compared to traditional methods, there are still some problems that limit its potential in this field: dataset scale, data imbalance, and domain shift. Besides, the manual annotation requirement to perform segmentation tasks is still a challenge, specially in large multi-site datasets. A possible solution would be the development of semi-supervised and unsupervised approaches, minimizing the usage of such labels. Overall, current issues related to poor reproducibility, specially for images from different domains (sites), impact the further applicability of DL models in a clinical environment.

### 4.2. Classification

The usage of multi-domain data for brain disease classification has recently attracted increasing attention because many participants from multiple domains could be beneficial for investigating the pathological changes in brain MR imaging, improving the results reliability and generalization capability. Although several DL methods have been developed, they usually assume that the source classifier can be directly transferred to the target domain (*i.e*., mapped between domains). Such methods used domain-invariant features, ignoring the domain shift in data distributions.

Kondrateva et al. (2021) analyzed and compared different MR imaging studies that tackle the domain-shift problem. They focused on advanced data processing, auto-encoding neural networks and their domain-invariant variations, model architecture enhancing, and feature training, as well as a prediction in a domain-invariant latent space approaches. They discovered that data augmentation before training (a conventional for most segmentation models for MR imaging) was the most widely used approach. Other commonly used approaches included adaptive receptive fields (*i.e*., deformable convolutions), as well as specialized loss functions. Authors also highlighted domain-invariant auto-encoding models that were found to be the most rapidly developing approach in medical segmentation for addressing the domain-shift problem. Key limitations included data normalization techniques like *z*-normalization and histogram matching (conventionally used in many MR imaging studies). Another limitation was related to the usage of DL-style transfer in CT and MR imaging, that was found to be unstable.

A simpler approach to reducing the impact of domain shift caused by varying acquisition parameters and processing pipelines was proposed by Hofer et al. (2017). Their proposed approach performed adaptation in feature space directly to overcome the deleterious effects of domain shift. They evaluated their approach using volumetric features to distinguish neurodegenerative diseases and reported results using three different datasets. They compared two different scenarios: (1) multi-site learning and (2) the use of pre-trained classifiers across different datasets. The proposed adaptation techniques for both scenarios performed similarly to the single-data case. While the proposed model was simple and can be easily estimated, in applications that the data cannot be modeled using Gaussian distributions, additional complexity may be required.

Islam and Zhang (2018) proposed a classification considering data variability applied to AD diagnosis, using an ensemble of deep CNNs. Instead of implementing a binary classification problem, they proposed to identify different stages of AD and obtained superior performance for diagnosing early-stage disease. They conducted experiments to demonstrate that their proposed model outperformed comparative baselines on the OASIS dataset (Fillmore et al., 2015). Even though their proposed model has been tested only on AD, the translation to other medical imaging classification is expected to be straightforward, showing potential for application in areas with a limited dataset.

Another AD classification approach was proposed by Jain et al. (2019) based on transfer learning (using VGG16 Simonyan and Zisserman, 2015 trained on ImageNet dataset Deng et al., 2009 as the feature extractor). The authors, using the ADNI dataset (Wyman et al., 2013), demonstrated that even though VGG16 was trained on general images (natural images), it was still able to extract useful features to support the AD brain MR imaging classification task. The model compared favorably to other classification model. In future studies, Jain et al. (2019) will investigate the removal of pre-processing steps, such as skull striping and intensity normalization, and fine-tuning the pre-training convolutional layers of the base model to improve performance.

Finally, Zhang et al. (2019) used structural brain MR imaging with an unsupervised model for brain disease identification. An unsupervised conditional consensus adversarial network (UCAN) for deep domain adaptation was used to learn a disease classifier trained from labeled source domain data and then adapt it to a different unlabeled target domain. The proposed model has three main components: (1) a module to extract features from the input MR imaging, (2) a module to perform cycle feature adaptation, supporting feature and classifier adaptation between the two domains (source and target), and (3) a classifier for disease identification. Experiments were performed on the ADNI dataset (Wyman et al., 2013) to analyze the effectiveness of the UCAN method compared to related approaches, showing better performance. In the experiments, the three orthogonal image views were applied to generate a prediction for each patient, using majority voting to combine the three results. The best performance was achieved by using only the sagittal view. This study reports the first use of a feature extraction module to learn representations to input MR imaging, followed by a cycle feature adaptation module to harmonize features and classifiers of the source and target domains. Zhang et al. (2019) suggested as relevant future work to use three-dimensional (3D) convolution as opposed to the computational easier two-dimensional (2D) convolution, taking advantage of the global structure information of 3D MR image volumes.

The work presented in this section aims to minimize the usage of pre-processing techniques, developing more complex models to tackle batch effects related to varying acquisition parameters in multi-site data, using domain adaptation strategies. Most of the classification approaches on multi-site data rely on accessing labeled data in all domains (source and target) when training the models, however, labels in target data may not be available. So in future works, there is a requirement for the development of DL models (Song et al., 2021), specially domain adaptation strategies to tackle data variability, applied for brain MR imaging using less manual annotation.

## 5. Discussion

We reviewed the literature describing the development and evaluation of DL models in human brain structural MR imaging (section 2). We first focused on issues related to data access, including summarizing key, broadly used, public datasets, and discussing emerging ethical and privacy issues. DL models that employed multi-site datasets were organized into approaches that use either: (1) Data Harmonization or (2) Domain Adaptation strategies. In the first approach, data harmonization (section 3), the impact of batch effects is reduced by standardizing/harmonizing images or by minimizing acquisition-based image variation. Domain adaptation (section 4), in contrast, employed models that implicitly handle batch effects resulting from different data sources and processing pipelines. For both data harmonization and domain adaptation approaches, we then described key advanced for both segmentation and classification implementations. In general, these methods provide either (1) predictions related to the occurrence of diseases or (2) analyze brain structures, typically in both patients with brain pathology and HC participants.

While there are numerous studies describing segmentation and classification tasks that use brain MR images, one of the main challenges in this area is the successful translation of these techniques to clinical practice. A re-occurring issue frequently preventing clinical adoption relates to poor capacity for generalization with many of the proposed models. Often this limitation is due to a poor management of variability inherent to different datasets. We identified batch effect and used it to describe variability that if improperly managed contributed to poor model generalization.

### 5.1. Identified Major Challenges

We identified major challenges when developing DL models and multi-site brain structural MR datasets. These challenges fall into four categories: (1) difficulties with identifying the appropriate literature, (2) problems with accessing appropriate datasets, (3) a general lack of annotation of large datasets, and (4) understanding the inherent trade-off between data harmonization and domain adaptation approaches.

One of the initial challenges when reviewing the literature was selecting appropriate search terms and keywords. We found multiple terms and field-specific jargon that were used to define the same concepts, *e.g*., dataset bias, domain shift, domain adaptation, *vs*. domain transfer. In practice, we found little standardization in the literature, making it challenging to identify the appropriate relevant studies.

Data access issues included the observation that many studies did not exclusively use publicly available datasets, but at times relied on private (proprietary) datasets. This observation poses a difficulty when trying to replicate findings. A further challenge when working with brain structural MR images is the limitation of accessing relative small volumes of data. Data augmentation approaches that realistically mimic variations in brain MR imaging data can alleviate the need for larger datasets, and transfer learning could be used to facilitate the training by using other already well-performing DL models (trained with HC participants and/or patient brain MR imaging data). Transfer learning in general improved the generalization of these models across datasets with less effort than learning from scratch (Akkus et al., 2017). Even after using these and other similar strategies, the limitation of small datasets remains a concern. Related somewhat to data size limitations are the development of models that can process 3D (or volumetric) data. The implementation of 3D models were generally restricted to 2D realizations where a series of image slices were processed (Swati et al., 2019). Such 2D models take advantage of the many image slices per image volume to increase the apparent data size. However, 2D implementations lose the volumetric information that may be relevant to the task of interest. Data unbalance and the presence of poor quality samples (*i.e*., image noise data, image artifacts such as from participant motion) also represent challenges in the development of reliable DL approaches.

The absence or poor data annotation (or labeling) represents a key challenge in the development of supervised DL models. DL models that are highly robust to variations in the labeling quality of brain MR imaging or have unsupervised learning capability that lessen the requirement on labels are needed (Sajja et al., 2006). Retraining a supervised model with data from each new domain is not feasible, simply because it requires manual annotation from experts (Ackaouy et al., 2020). Self-learning and unsupervised learning are promising models that may be used to overcome limitations related to poor or missing data annotation.

An advantage to more advanced models is that they can explicitly handle batch effects, thus presenting consistent results over different datasets (Yan et al., 2020). This trade off is reflected in general between simpler Data Harmonizing methods *vs*. the more complex domain adaptation methods that implicitly account for variability. However, the development of the more complex models may impose another challenge that is related to decreasing model interpretability (Yosinski et al., 2015; Yamashita et al., 2018). In most situations, these models are considered to be “black boxes,” potentially limiting their application in the clinical environment (Ho et al., 2019). However, there are techniques that can overcome this limitation and provide further interpretation and understanding of more advanced DL models. Layer-wise relevance propagation, for example, is one technique that maps an abstract concept (*e.g*., features associated with a predicted class) into a domain that a human observer can visualize (Montavon et al., 2018).

The translation from models trained on one brain pathology to other brain pathologies is another challenge in this area. There is a specific effort to avoid using application-related information when designing the DL models, however, it is still not clear how well the findings of specific studies can generalize to the studies of other brain diseases (Tohka et al., 2016). This unknown is highly relevant and needs to be considered in the translation to clinical practices.

### 5.2. Recommendation for Future Studies

Future studies should extend the evaluation of DL approaches to structural MR data that were not present during the initial training phase (Karani et al., 2018). Application-specific DL models are emerging, and new methods are continuously explored and introduced. The selection of the most appropriate technique for a given application is a difficult task. In many cases, a combination of several techniques may be necessary to obtain reliable and generalizable performance. Domain adaptation approaches that employ transfer learning are preferred to training models from scratch (Ghafoorian et al., 2017). Commonly, the integration of different datasets (acquired from different research centers or scanners, or over time), if appropriately processed, can help to improve the model performance, presenting consistent results when compared with models developed using single-site data. The study of DL models developed using multi-site data, specifically the understanding of variability and bias effects, is an initial step to help translate medical image DL applications to the clinical setting (Despotovic et al., 2015).

Current state-of-the-art work in this area are focused on domain-adaptation techniques, mostly using adversarial approaches. While a pure adversarial setting may be ineffective (Orbes-Arteaga et al., 2019), improved performance across all models was obtained when combining it with other task-specific strategies.

A possible approach to handle classification or segmentation tasks on structural brain MR is to perform a fully 3D network, and using adversarial discriminative networks (Dewey et al., 2019). This method presented great results to accomplish classification studies, however, it requires retraining on the target domain images. This finding is a limitation when translating to the clinic, where acquisition parameters can change frequently.

There are key studies advancing domain adaptation methods including the development of new optimized strategies that could be translated to neuroimaging. Ajakan et al. (2014) proposed a representation learning algorithm to perform domain adaptation. Their model is trained to perform a classification task, while minimizing the influence of the domain the images were acquired from. Experiments performed using unlabeled target domain data (minimizing the requirements for data annotation), had superior results when compared to standard neural networks or a support vector machine approaches.

Zhao et al. (2019) developed an unsupervised domain adaptation approach using classification and regression settings, aiming to present reliable results in multiple domains. The proposed multi-source domain adversarial network (MDAN) is not limited to single source and single target domain applications as other domain adaptation approaches. The authors presented improved findings in non-medical imaging applications like sentiment analysis, digit classification, and vehicle counting. Further, these and similar strategies present key required characteristics for medical imaging applications, such as minimizing the usage of manual annotation (unsupervised approaches or unlabeled target data domain), and present reliable results across multiple domains found in clinical environments and clinical trials. The translation of domain adaptation models like MDAN for the development of DL tools using structural brain MR may overcome the current challenges in this area, specifically in multi-site studies.

Another suggestion for future work is to explore multi-channel CNNs or a cascade of CNNs working in parallel with individual image sequences. The individual outputs could then be combined using a customized bagging strategy (Jollans et al., 2019). The usage of multiple data points from individual study participants provides new and potentially complementary information to perform classification and segmentation tasks.

The combination of data harmonization (to eliminate poor quality data and explicitly minimize batch effects by standardizing data) followed by application of domain adaptation strategies (to implicitly manage batch effects) may be the best solution to improve model reliability and reproducibility in large heterogeneous studies (see solid line in Figure 3). Such approaches warrant further investigation.

Another pertinent topic in the suitability of DL models for human data across diverse populations. Issues of model bias and fairness can result from datasets lacking diversity. Many current studies fortunately enhance data diversity by including both male and female participants over a wide age range. Other studies include HC and patient participants. Future studies must consider, identify and minimize population bias and, thus, assure non-discriminatory decision making.

Although DL greatly improves the performance of automatic computer-based methods, it is still challenging to directly use in clinical analysis. MR images can have highly variable characteristics across patients, MR imaging scanners, and imaging protocols. Collecting large-scale datasets to tap the potential of DL can help accelerate its application in clinical medicine, but there is still a lot of room for improvement for such methods. The method with better performance and stronger robustness are undoubtedly beneficial to the doctor's pre-diagnosis and post-treatment of the patient's condition (Zeng et al., 2020).

## 6. Conclusion

MR is a flexible medical imaging modality, but often lacks reproducibility or consistency between protocols and scanners. Hence, early diagnosis of brain disease using computer-aided systems, while of great importance and extensive research amongst researchers, is challenging. DL-based approach is garnering much attention, primarily due to its state-of-the-art performances in a variety of classification and segmentation tasks. Several recent studies, that have used brain MR images with DL architectures, have shown promising results for in several brain diseases and disorders. However, the most common issue when using DL architectures like CNN is that they require a large amount of data for training. Aggregating or combining data across different scanners, should prove even better at identifying scanner effects as between-scanner batch effects is generally much larger than within-scanner variability (Li et al., 2020), and thus improve generalization. While many datasets are publicly available, most algorithms are still trained on a single dataset and often suffer the problem of limited sample sizes and are adversely impacted by data unbalanced.

DL methods have demonstrated good performance in medical image analysis. Yet only a few applications have successfully being translated and are now in clinical use. One of the reasons for this poor translation is the poor understanding of batch effects. We have reviewed the development of DL models that were trained with data acquired on different scanners or on the same scanners but with different acquisition parameters. Addressing these differences across these studies is important to evaluate the feasibility of using such models on different datasets, and improving eventual translation to the clinical environment.

We have summarized DL models as they are applied to large and heterogeneous brain imaging datasets by examining: data access, data harmonization, and domain adaptation methods. We discussed the emerging, more complex, DL models that have been developed to implicitly handle data variability and presented the main challenges in this research area. Domain-adaptation strategies, such as using adversarial networks, were showed to be one of the main strategies to address the effects of bias and variability on multi-site brain MR imaging datasets.

## Author Contributions

MB, JP, and IF performed the literature review and screening papers according to the inclusion criteria (presented in the paper). LR and RF wrote part of the discussion and the recommendation for future studies sections (critical sections in a review paper). MB organized the selected papers, categorized them into sections (according to similarities), and wrote the first draft of the manuscript. All authors summarized the currently available public datasets, performed manuscript revision, and approved the submitted version.

## Funding

This study was partially funded by the Canadian Institute for Health Research (MOP-333931) and the Brazilian Institute of Neuroscience and Neurotechnology (BRAINN) network (funded by the São Paulo Research Foundation FAPESP—process CEPID 2013/07559-3). Infrastructure supporting some of our studies was provided by the Canadian Foundation for Innovation. Key aspects of the Calgary-Campinas collaboration were initiated in 2014 by a grant from the Brazilian Office of Coordination for the Improvement of Higher Education Personnel (CAPES, PVE 88881.062158/2014-01). MB received post-doctoral fellowship funding from the Hotchkiss Brain Institute at the University of Calgary, and from Brain Canada through the Canadian Open Neuroscience Program (CONP). RF is the Hopewell Professor of Brain Imaging at the University of Calgary.

## Conflict of Interest

The authors declare that the research was conducted in the absence of any commercial or financial relationships that could be construed as a potential conflict of interest.

## Publisher's Note

All claims expressed in this article are solely those of the authors and do not necessarily represent those of their affiliated organizations, or those of the publisher, the editors and the reviewers. Any product that may be evaluated in this article, or claim that may be made by its manufacturer, is not guaranteed or endorsed by the publisher.
